# Transvaginal single-pathway composite reconstruction for pelvic organ prolapse combined with rectal prolapse: a case report

**DOI:** 10.3389/fsurg.2026.1873845

**Published:** 2026-07-21

**Authors:** Xi Luo, Na Yan, Neng Jing Yin, Ting Liu, Guangshi Tao, Li Sha Zou

**Affiliations:** 1 Department of Gynecology, The First Affiliated Hospital of Hunan Traditional Chinese Medical College (Hunan Provincial Directly Affiliated Hospital of Traditional Chinese Medicine), Zhuzhou, China; 2 Department of Medical Ultrasonics, The First Affiliated Hospital of Hunan Traditional Chinese Medical College (Hunan Provincial Directly Affiliated Hospital of Traditional Chinese Medicine), Zhuzhou, China; 3 Department of Gynecology, The Second Xiang ya Hospital of Central South University, Changsha, China

**Keywords:** case report, pelvic organ prolapse, rectal prolapse, surgery, transvaginal mesh

## Abstract

Concomitant pelvic organ prolapse (POP) and rectal prolapse (RP) is a relatively rare and often underrecognized clinical condition, typically managed as two separate entities within a single-disciplinary framework. Historically, transvaginal mesh (TVM), although widely used in pelvic floor reconstruction, has not been applied to treat RP. We report a 76-year-old female with severe POP and concomitant RP. Guided by the integral theory of pelvic floor, we innovatively cut and assembled TiLOOP Total 4 mesh into Total 6 configuration, connected it to a trimmed TiLOOP mesh (10 × 15 cm), and performed transvaginal fixation of the anterior rectal wall using the mesh during TVM pelvic floor reconstruction. This modified TVM pelvic floor reconstruction with concurrent TVM rectopexy achieved unexpectedly favorable outcomes, with no recurrence or mesh-related complications at 18 months of follow-up.Our findings suggest that, in selected cases meeting the indications, combined TVM pelvic floor reconstruction with concurrent TVM rectopexy appears to be feasible and may represent a potential effective treatment option for colorectal and gynecologic surgeons managing concurrent POP and RP. It should be noted, however, that this is a single case report with limited sample size, no control group, and short-term follow-up. Future studies with cohort designs or extended follow-up are warranted to further validate the efficacy of this procedure.

## 1 Introduction

Pelvic organ prolapse (POP) affects 10% of the female population (1). It is defined by vaginal wall descent to or beyond the hymen on maximal Valsalva (2),. POP, includes anterior vaginal wall descent（79%）, as well as uterine prolapse（49%） and posterior vaginal wall descent (31%) (3). Rectocele is defined as the protrusion of the rectal wall together with the posterior vaginal wall (PVW) toward the vagina (4), and it almost invariably coexists with posterior vaginal wall prolapse (5).Rectal prolapse (RP) is a benign protrusion of the rectal mucosa through the anus, with a reported prevalence of 0.5% (6). Concomitant POP and RP are uncommon(2.0% of POP patients) and often clinically overlooked (7). Traditionally, such patients required multidisciplinary coordination, separate surgical approaches. Whereas Simultaneous surgical correction not increase complication rates (8), and such combined procedures are increasingly performed. Current combined approaches—abdominal sacrocolpopexy with ventral mesh rectopexy, transvaginal hysterectomy with rectopexy, or perineal Altemeier/Delorme procedures with colpocleisis (9). These surgeries, however, are largely based on traditional, discipline-specific concepts of pelvic floor reconstruction, focusing on isolated anatomical defects while neglecting the integral nature of the pelvic floor. There remains a lack of a single-route surgical strategy based on the holistic concept of the pelvic floor for managing complex multi-compartment prolapse.

In April 2019, the U.S. FDA banned the use of transvaginal mesh (TVM) for POP due to risks of mesh erosion, exposure, and pain (10). However, TVM, grounded in the holistic concept of the pelvic floor, offers a high anatomical cure rate (90%) (11), minimal invasiveness, and particular suitability for elderly or medically frail patients with recurrent or complex multi-compartment severe prolapse. As such, it remains an important treatment option. International authoritative organizations, including FIGO (12) and NICE, have subsequently issued clinical recommendations for its use. In China, TVM is indicated for recurrent POP or primary severe POP in women aged ≥60 years (13). Moreover, Complications are largely associated with early-generation meshes (14). Lightweight mesh systems and novel biomaterials have substantially reduced (15, 16) Thus, TVM-based pelvic floor reconstruction remains a viable option under selective and individualized considerations.

**Figure 1:**
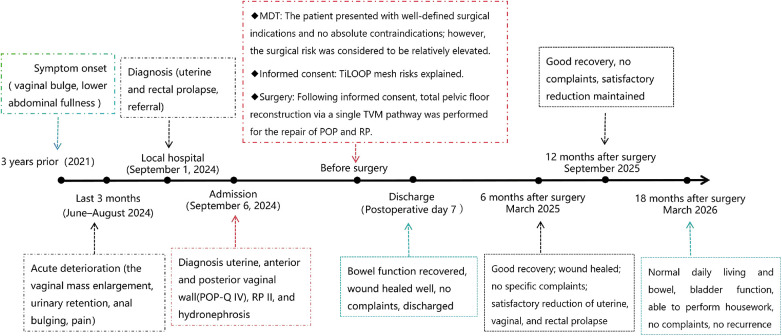
Time line.

In this case, we performed TVM pelvic floor reconstruction, suspending the anterior rectal wall by trimming the pelvic floor repair mesh from TiLOOP Total 4 to Total 6 and splicing it with TiLOOP Mesh. The procedure showed remarkable efficacy, and no recurrence or postoperative complications were observed at 18 months of follow-up. The timeline of the case is shown in Figure 1. This case suggests that, in specific situations, TVM may be a useful option for anorectal surgeons and gynecologists treating POP combined with RP, and we report this case for clinical reference and further exploration.

## 2 Case description

### 2.1 Clinical findings

The patient is a 76-year-old female farmer who presented with a vaginal prolapse for 3 years that had worsened over the past 3 months.

Three years earlier she first noticed a vaginal bulge about the size of an egg, associated with lower abdominal fullness. Symptoms worsened with physical activity and improved with rest. She reported no vaginal bleeding and no stress-related urine leakage, but she did have urinary incontinence. She has chronic constipation requiring laxatives and her symptoms progressively worsened with prolonged heavy lifting. Over the last 3 months the vaginal mass markedly enlarged and she developed urinary retention requiring catheterization, as well as anal bulging and pain. She presented to a local hospital where she was diagnosed with rectal and uterine prolapse and advised to seek surgical treatment at a higher-level center. She was referred to Hunan Provincial Hospital of Traditional Chinese Medicine for more comprehensive evaluation and management. Medical, family, and psycho-social history were unremarkable.

### 2.2 Diagnostic assessment

#### 2.2.1 Gynecological examination

**Figure 2:**
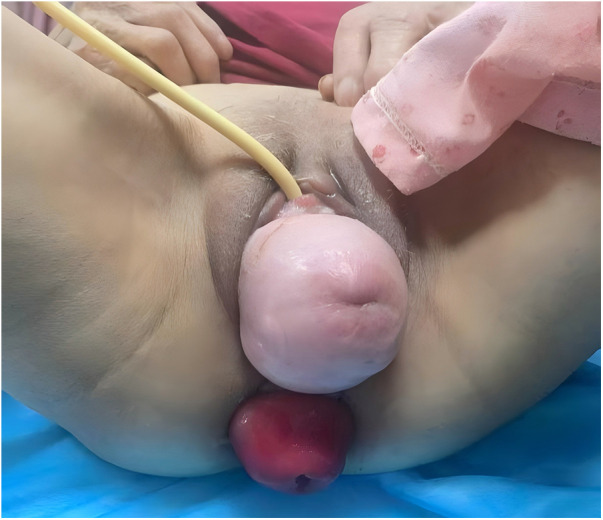
Preoperative POP Status(indwelling urinary catheter. uterine prolapse.rectal prolapse).

The vulva appeared consistent with age-related changes, showing no signs of leukoplakia or ulceration. The vaginal mucosa was smooth, with no abnormal secretions observed. The perineum exhibited an old second-degree fissure. Additionally, an old second-degree laceration was noted in the perineum, and a prolapsed anal mass measuring approximately 4 cm in length was observed. The findings of the gynecological examination are shown in Figure 2.

#### 2.2.2 Ask the patient to perform a bowel movement anus

**Figure 3:**
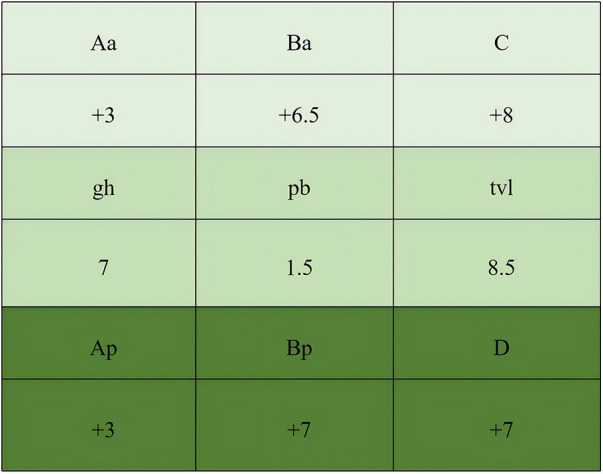
POP-Q score (Aa: reference point on the anterior vaginal wall located 3 cm proximal to the hymen (range –3 to +3). Ba: the most distal point on the upper anterior vaginal wall (≥ Aa). Ap: reference point on the posterior vaginal wall located 3 cm proximal to the hymen (range –3 to +3). Bp: the most distal point on the upper posterior vaginal wall (≥ Ap).C: the most distal edge of the cervix or vaginal apex. D: the posterior fornix behind the cervix. gh: Genital hiatus length.PB: Perineal body length.TVL: Total vaginal length.).

The anal mucosa and basal layer prolapsed 6 cm beyond the anal opening, forming a spiral shape. The mucosa appeared bright red. Vagina: The anterior vaginal wall (associated with the bladder) and the posterior vaginal wall were completely detached from the external vaginal opening. Cervix: The cervix and the uterine body have prolapsed beyond the vaginal opening, with the C-point approximately 8 cm from the hymenal margin. There is no mucosal breakage. Refer to Figure 3 for the pelvic organ prolapse Quantification(POP-Q) score.

#### 2.2.3 Bimanual examination

The uterine body was found to be atrophic, exhibiting a moderate degree of consistency and no tenderness. Bilateral adnexal regions: No palpable masses or tenderness were identified.

#### 2.2.4 Digital rectal examination and anoscopy

The anal sphincter exhibited laxity, while the rectal mucosa protruded into the anal canal no palpable masses were detected.

#### 2.2.5 Transvaginal four-dimensional color Doppler ultrasound of the pelvic floor

The patient exhibited a marked cystocele (Type III), uterine prolapse, and RP, as evidenced by imaging. The patient exhibited excessive movement of the perineal body and severe dilation of the levator ani muscle foramen. However, there was no evidence of rupture of the levator ani muscle or anal sphincter on imaging. The patient exhibited symptoms of urinary retention, characterized by a residual urine volume of 200 mL. The whole-abdominal Computed Tomography(CT) scan revealed dilatation of the entire left ureter, mild hydro nephrosis of the left kidney, small stones in both kidneys, and intestinal distension.

#### 2.2.6 Computed tomography urography Ctu

1. The condition under consideration is uterine prolapse, which is characterised by the protrusion of part of the uterus outside the vagina. The second condition is dilatation of the left ureter and hydronephrosis of the left kidney. The aetiology of this condition is not clear, but it is possible that it is due to the lower segment of the ureter being compressed by the prolapsed uterus. 3. It was established that there were no abnormalities in renal excretory function. Furthermore, significant intestinal contents were observed in the colon and rectum.

#### 2.2.7 Clinical diagnosis

1. Uterine prolapse (POP-Q IV degree) 2. Anterior vaginal wall prolapse (POP-Q IV degree) 3. Posterior vaginal wall prolapse (POP-Q IV degree) 4. Rectal prolapse (II degree) 5. Old perineal laceration 6. Left-sided ureteral dilatation 7. Emaciation 8. Malnutrition 9. Spinal kyphosis 10. Urinary retention.

#### 2.2.8 Multi-Disciplinary treatment Mdt

Prior to the operation, a multidisciplinary team from the anorectal, anesthesiology, and urology departments convened. They determined that the patient's left hydronephrosis and urinary retention resulted from uterine prolapse compressing the ureter. Surgical intervention was indicated, and no contraindications were identified.

The Department of Anus and Gastroenterology diagnosed the patient with RP (II degree). The specialist recommended performing uterine prolapse surgery first, followed by RP surgery after recovery, necessitating two separate procedures. However, due to the patient's physical condition and financial constraints, she requested that both surgeries be performed simultaneously.

#### 2.2.9 Ethics approval and informed consent

The study was performed in accordance with the Declaration of Helsinki. The use of TVM was discussed with the patient, and she was specifically informed about its potential complications, including mesh exposure, erosion, and pain. Alternative treatment options were also presented. The patient provided written informed consent for the surgery and for the publication of this case report.

### 2.3 Therapeutic intervention

#### 2.3.1 Surgical strategy

The TiLOOP Total 4 mesh was divided into six sections(T6) for TVM, and it was cut and spliced with additional TiLOOP Mesh for anterior rectal wall fixation.

#### 2.3.2 Mesh cutting and splicing

**Figure 4:**
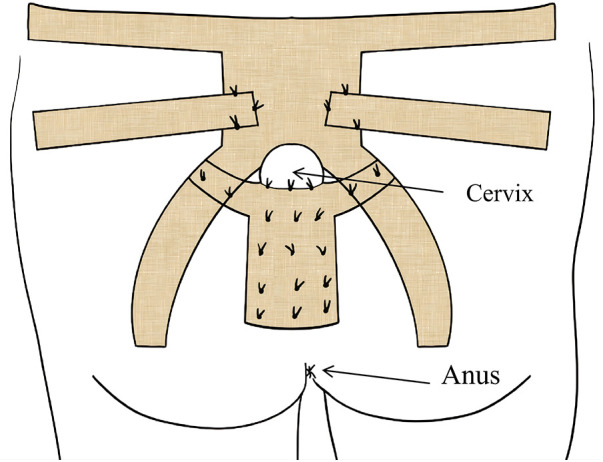
Diagram of mesh cutting and assembly（From a 10 × 15 cm mesh patch, two arms were cut to secure the tiLOOP total 4 mesh, thereby splicing it into a tiLOOP total 6 mesh.the residual mesh was trimmed as depicted for rectal suspension. The figure shows the fixation knots of the mesh and the rectal suspension.).

The TiLOOP Total 4, was cut and combined with the 10 × 15 TiLOOP mesh to form three fixation straps on each side (Total 6: Total pelvic floor reconstruction). The central part of the mesh has an anterior-posterior diameter of 7 cm. The remaining mesh was cut into a 4 × 15 cm piece, with its upper end trimmed into a “U” shape, with each side arm measuring approximately 3 cm in length. The remaining mesh measured 12 cm in length and 4 cm in width, and will be used for fixation and suspension of the anterior rectal wall. For more information, please refer to Figure 4.

Special curved puncture needles were employed to extract the six slings. The anterior pelvic mesh was placed tension-free and flat in front of the bladder. The lower edge of the 3-0 suture was secured to the main ligament of the cervical ring, while the upper edge of the 0# suture suspended the bladder fascia. The edges of the vaginal wall were trimmed, and the incision was closed with a 2-0 absorbable thread. The posterior pelvic mesh was positioned flat in front of the rectum. Two to three stitches were made to the levator muscle, and 3-0 sutures were used to attach the mesh's corners to the rectal fascia. Finally, the posterior wall was closed with 2-0 absorbable sutures.

#### 2.3.3 Mark the skin puncture points and make incisions as follows

For the anterior pelvis, make an anterior incision at the urethral orifice level, 0.5 cm lateral to the subpubic branch. The posterior incision should be 1 cm lateral and 2 cm inferior to the anterior incision. For the posterior pelvis, mark 3 cm posterior to the outer edge of the anus. Use a No. 11 blade to make a 0.5 cm skin incision.

#### 2.3.4 Puncture fixation involves three branches

Upper, middle, and lower. For the upper branch, the puncture needle is inserted around the pubic arch through the pelvic fascia tendon arch, guiding the upper branch fixation band through the puncture site. For the middle branch, the needle penetrates the pelvic fascia tendon arch at the superior border of the sciatic spine, and the middle branch is secured by threading it through the puncture site. For the lower branch, the needle passes through the middle part of the sacrospinous ligament, and the middle branch is similarly secured by threading it through the puncture site.

In rectal reconstruction, the mesh is secured at the upper end to the sacrospinous ligament fixation band on each side. Centrally, it is attached to the deep muscular layer of the posterior cervical vault. The mesh body is sutured to one-third of the muscular layer of the anterior rectal wall. The lower end of the mesh is fixed to the inner vaginal mucosa at the junction between the lower end of the vagina and the rectum. For further details, refer to Supplementary Video 1.

### 2.4 Outcome

**Figure 5:**
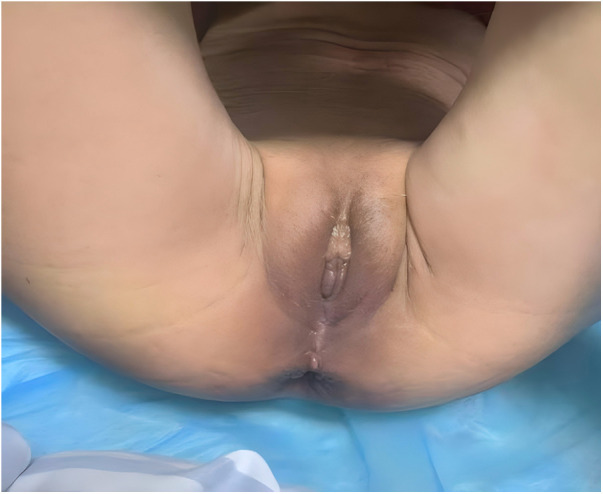
One week post-surgery perineal Status.

Through detailed preoperative evaluation, precise surgical technique, and careful postoperative care, the rectum was packed with Vaseline gauze rolls wrapped in standard imitation gauze to provide compression and hemostasis and to limit fecal contamination of the operative field. Postoperatively, intravenous nutritional support was intensified and traditional Chinese medicine(TCM) was administered to promote rapid recovery. The patient recovered fully and was discharged one week after surgery. the condition at discharge is detailed in Figure 5.

### 2.5 Postoperative follow-up

**Figure 6:**
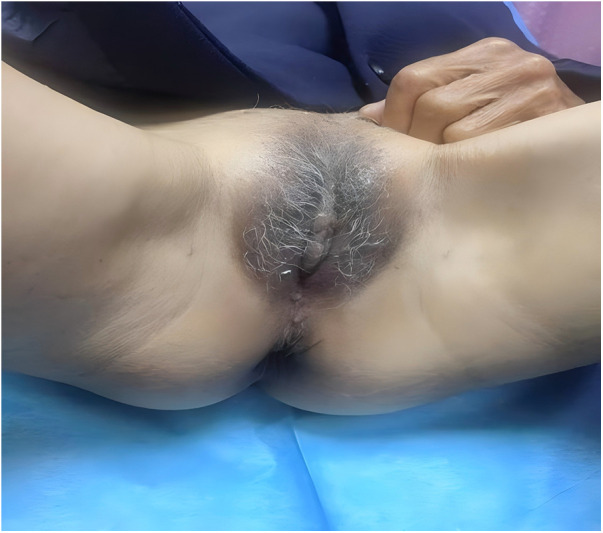
12-Month post-surgery perineal Status.

Postoperative follow-up was conducted at 6 months and 12 months in the outpatient clinic, and at 18 months via telephone. At the 6- and 12-month clinic visits, pelvic examination with POP-Q assessment revealed no recurrent prolapse (stage 0 for all compartments). No mesh exposure, erosion, or pain was observed. The patient reported normal urination without catheterization and resolution of anal bulging. At the 18-month telephone follow-up, the patient reported sustained normal urination, resolution of anal bulging, and full return to daily activities including household tasks. No objective POP-Q or imaging assessment was available at the 18-month telephone follow-up, which is a limitation of this report. See Figure 6 for the perineal status at 12 months post-surgery.

## 3 Discussions

POP occurs when pelvic organs descend from their normal anatomical position along the vagina. Surgery becomes the primary treatment option when the POP-Q score reaches degree III to IV. Advances in surgical techniques, materials, and concepts have expanded treatment options. These range from traditional transvaginal repair to transabdominal or transperitoneal laparoscopic surgery, and now include the use of synthetic materials like total pelvic floor reconstruction with TVM. Surgical approaches continue to evolve, focusing on individualized treatment to enhance cure rates, reduce postoperative recurrence, and minimize complications. With the advancement of modern anatomical theories, surgical approaches for POP have evolved. The traditional method of simple excision, fixation, and suturing has gradually transitioned to techniques focused on repairing anatomical defects in the pelvic floor. These newer methods aim to restore the biomechanical function of tissues, effectively use alternative materials, and minimize trauma to achieve therapeutic goals.

The holistic theory of the pelvic floor conceptualizes the pelvic floor as an indivisible functional unit, focusing on pelvic floor dysfunction caused by laxity of muscles, fascia, and ligaments. This theory provides an interdisciplinary framework for research, diagnosis, and treatment, substantially advancing systematic innovation in the field. Total pelvic floor reconstruction with TVM aligns well with the structural principles of the pelvic floor. It is particularly suitable for severe (stage III–IV) prolapse when curative effects are pursued, offering a very low recurrence rate (5%–8%), long-lasting efficacy, minimal invasiveness, and rapid recovery. Owing to reported complication rates of mesh exposure and erosion (approximately 1.3%–8%), the U.S. FDA imposed a ban on TVM sales in April 2019 (17). However, that ban was largely based on complications associated with early-generation meshes**,** and TVM use for POP remains controversial.With the recent introduction of lightweight mesh systems (18) and novel materials (e.g., PVDF), clinical outcomes have reportedly improved. A 2025 nationwide Finnish study with a median follow-up of 7.4 years reported a reoperation rate of 10.1% in the TVM group versus 13.9% in the native tissue repair group (*p* = 0.09), a clinically meaningful difference, with TVM demonstrating significantly lower recurrence rates during certain follow-up intervals. Nevertheless, long-term data for newer meshes remain limited. Pelvic floor reconstruction should, in principle, be individualized. On June 23, 2025, the Chinese Clinical Application Standards for Transvaginal Mesh Implantation in Pelvic Floor Reconstruction for Pelvic Organ Prolapse were issued (13). Unlike the FDA ban, this document explicitly defines the indications, contraindications, and clinical practice standards for TVM use in China, thereby establishing a legal basis for its conditional clinical application under strict indications and standardized procedures.

A nationwide, prospective, longitudinal registry study covering 103 tertiary referral centers in China included 5,621 patients who underwent TVM surgery between July 2019 and December 2022. The results showed an intraoperative complication rate of 3.9%, and mesh exposure occurred in 83 cases (1.48%), of which 76 had an area <1 cm^2^ and 46 were asymptomatic. At two-year follow-up, the anatomical cure rate was 91.9%, the subjective success rate was 93.3%, and patient satisfaction was 98.2%. The study concluded that TVM repair yields satisfactory anatomical and subjective outcomes with a low mesh exposure rate (19). Separately, another study found that adding a graft or mesh during posterior vaginal repair did not significantly improve outcomes; however, this “no benefit” finding applies to routine use in primary, isolated rectocele and does not exclude high-risk scenarios such as recurrent, severe, or multi-compartment prolapse (20). The 2024 French clinical practice guidelines recommend that mesh use be restricted to patients with recurrent prolapse or stage 3–4 prolapse, or those who provide informed consent to participate in prospective studies (21). Novel meshes [lightweight (22), PVDF (23), biologic] offer lower recurrence rates and acceptable complication profiles in high-risk populations. Under strictly defined indications (high recurrence risk, severe prolapse, multi-compartment involvement) and with adequate informed consent, transvaginal mesh remains a viable and evidence-supported reconstructive option.

RP is a benign condition where the rectal mucosa, anal canal, and sometimes part of the sigmoid colon descend and protrude outside the anus. It is generally classified into two types: incomplete and complete RP. In incomplete RP, only the rectal mucosa layer protrudes, forming a hemispherical shape with a ring-shaped mucosal groove centered around the rectal cavity. In complete RP, the prolapsed rectum is conical, with the mucosal ring groove arranged concentrically around the rectal cavity (24). The severity of prolapse is categorized into three degrees. Degree I involves the rectum forming an umbrella-like shadow in the jugular abdomen. Degree II involves the rectum protruding outside the anus, with normal anal sphincter function and anal canal position, and no anal incontinence. Degree III involves the rectum, part of the sigmoid colon, and the anal canal protruding outside the anus, with impaired anal sphincter function and incomplete or complete anal incontinence. Additionally, the internationally recognized Oxford Scale classifies RP into degrees I-V (25). Surgical procedures for RP are divided into two main categories: the transabdominal and transperineal approaches. The transabdominal approach includes posterior rectal suture fixation surgery (26), sigmoidectomy, transabdominal rectal suspension fixation surgery (27), and lateral rectal fixation surgery. The transperineal approach comprises transanal anastomotic rectal resection, transperineal rectal mucosa stripping and muscle folding surgery, anal tightening surgery, and transperineal rectosigmoidectomy.

The prevalence of POP is approximately 10%, whereas that of RP is about 0.5% (28). Although POP frequently coexists with rectocele, both POP and RP share the common pathogenesis of degeneration and damage of pelvic supporting structures; however, the coexistence of POP and RP is often overlooked. Umeh et al. (29) reported a 40-year-old woman who underwent open uterosacral and rectal fixation. Karateke et al. (30) described the management of uterine prolapse in a 77-year-old and an 83-year-old woman using vaginal colpocleisis combined with the Delorme and Thiel procedures for RP. Kaori Yamashita et al. reported an 80-year-old woman with concurrent uterine and rectal prolapse treated with a single posterior transvaginal mesh (TVM) procedure followed by a routine anterior TVM procedure under general anesthesia; nevertheless, the repair was still performed in two steps.

To our knowledge, report of RP repair via a transvaginal approach using TVM remain extremely limited. Using TVM to address both POP and RP is consistent with the holistic theory of the pelvic floor and adheres to the principle of individualization, particularly for elderly patients with severe POP and RP who cannot tolerate abdominal, laparoscopic, or repeated surgery. This approach also meets the indications outlined in Chinese clinical guidelines. A single-center, prospective cohort study from China evaluated the safety and long-term efficacy of transvaginal pelvic floor reconstruction in 343 elderly patients (aged ≥70 years) with severe POP (31). The results confirmed that, for elderly POP patients who desire vaginal preservation and are not candidates for colpocleisis, transvaginal reconstructive pelvic surgery (TVRPS) is safe, feasible, and durable, provided comprehensive preoperative evaluation and rigorous patient selection are performed (32). Furthermore, the composite surgical success rate of self-cut mesh procedures is non-inferior to that of commercial mesh kits using the same titanium-coated polypropylene mesh, while hospital costs are reduced by 40.4%. These findings suggest that self-cut mesh surgery may be beneficial for selected women with severe POP, particularly in low- and middle-income countries.

This case involves a severe pelvic organ prolapse combined with RP, requiring collaboration between gynecology and anorectal departments. Addressing uterine and RP simultaneously through a single pathway is crucial for effectively treating pelvic floor prolapse combined with RP. We repaired the RP using artificial mesh anterior rectal wall fixation via the transvaginal pathway during total pelvic floor reconstruction. After one and a half years of follow-up, the patient has shown good recovery without recurrence or postoperative complications, maintaining a normal lifestyle. This case suggests that, in carefully selected patients, transvaginal artificial mesh implantation may serve as a viable treatment option for anorectal surgeons and gynecologists managing POP combined with RP.

## 4 Conclusions

In conclusion, the simultaneous occurrence of pelvic floor prolapse and RP is uncommon. This case introduces an innovative approach by cutting Total 6 mesh from TiLOOP Total 4 and splicing it with additional TiLOOP Mesh. The procedure involves repairing RP by securing the anterior rectal wall with artificial mesh via a transvaginal pathway during total pelvic floor reconstruction. This method effectively addresses the complex issue of multiorgan prolapse in a single operation, significantly reducing both hospitalization and patient recovery time while maximizing economic benefits.Under stringent indications, the modified TVM total pelvic floor reconstruction with concurrent RP repair is feasible. For combined POP and RP, TVM offers a complementary alternative to mainstream approaches, tailored to different patient profiles. Treatment selection should integrate the patient's overall condition, functional expectations, and surgical tolerance, and be determined via shared decision-making with an experienced surgeon.

## 5 Patient perspective

A long time ago, I felt an egg-sized lump coming out of my vagina during farm work. Over time, it slowly got worse. Then recently, I couldn't urinate and needed a catheter. The local doctor said my womb and rectum had fallen out.

The big hospital suggested two separate surgeries, but I am old and weak. I asked them to fix everything at once. They listened. Now I can urinate normally and feel much better.

## Data Availability

The original contributions presented in the study are included in the article/Supplementary Material, further inquiries can be directed to the corresponding author.

## Supplementary material

Supplementary Video 1 Transvaginal mesh for rectal fixation.

## References

[1] Wang B Chen Y Zhu X Wang T Li M Huang Y. Global burden and trends of pelvic organ prolapse associated with aging women: an observational trend study from 1990 to 2019. Front Public Health. (2022) 10:975829. 10.3389/fpubh.2022.975829 36187690 PMC9521163

[2] Collins SA O’Shea M Dykes N Ramm O Edenfield A Shek KL. International urogynecological consultation: clinical definition of pelvic organ prolapse. Int Urogynecol J. (2021) 32(8):2011–2019. 10.1007/s00192-021-04875-y 34191102

[3] Payebto Zoua E Boulvain M Dällenbach P. The distribution of pelvic organ support defects in women undergoing pelvic organ prolapse surgery and compartment specific risk factors. Int Urogynecol J. (2022) 33(2):405–409. 10.1007/s00192-021-04826-7 33974095 PMC8803792

[4] Metrinsky YY Belousov AM Sizonenko NA Korzhuk MS Mulendeev SV. Rectocele in females: unresolved issues (review). Koloproktologia. (2025) 24(4):188–200. 10.33878/2073-7556-2025-24-4-188-200

[5] Radzinsky VE Orazov MR Mikhaleva LM Krestinin MV Lologaeva MS. Pathogenesis of vaginal prolapse with the formation of rectocele: a review. Gynecology. (2021) 23(6):601–604. 10.26442/20795696.2021.6.201287

[6] Bordeianou L Paquette I Johnson E Holubar SD Gaertner W Feingold DL. Clinical practice guidelines for the treatment of rectal prolapse. Dis Colon Rectum. (2017) 60(11):1121–1131. 10.1097/DCR.0000000000000889 28991074

[7] Speed JM Zhang CA Gurland B Enemchukwu E. Trends in the diagnosis and management of combined rectal and vaginal pelvic organ prolapse. Urology. (2021) 150:188–193. 10.1016/j.urology.2020.05.010 32439552

[8] Rahman S Wallace SL Spivak AR Hortian VA. Ways to repair multicompartment prolapse: decision-making and surgical approach. Clin Colon Rectal Surg. (2025) 38(6):375–383. 10.1055/s-0045-1804514 41040119 PMC12488238

[9] Botoncea M Molnar C Nicolescu CL Cosma CD Butiurca VO Molnar DC. Concurrent pelvic organ and rectal prolapse: a narrative review of surgical perspectives. Chirurgia (Bucur). (2025) 120(5):502–510. 10.21614/chirurgia.3210 41196076

[10] Ray S Clifton M Koo K. MP05-10 accuracy of Media coverage about the 2019 US food and drug administration ban on transvaginal mesh for pelvic organ prolapse. J Urol. (2020) 203(Suppl 4). 10.1097/ju.0000000000000819.010 32439554

[11] Lasri S Alshamsi H Campeau L. Synthetic meshes in pelvic organ prolapse: a narrative review. Société Internationale d’Urologie Journal. (2025) 6(1):2. 10.3390/siuj6010002

[12] Ugianskiene A Davila GW Su TH. FIGO urogynecology and pelvic floor committee. FIGO review of statements on use of synthetic mesh for pelvic organ prolapse and stress urinary incontinence. Int J Gynaecol Obstet. (2019) 147(2):147–155. 10.1002/ijgo.12932 31353463

[13] Guangdong Medical Device Management Association. T/GDMDMA 0046-2025: Chinese clinical application standard of reconstructive pelvic surgery using transvaginal mesh implantation in the treatment of pelvic organ prolapse. Published June 23, 2025).

[14] Barski D Deng DY. Management of mesh complications after SUI and POP repair: review and analysis of the current literature. Biomed Res Int. (2015) 2015:831285. 10.1155/2015/831285 25973425 PMC4418012

[15] Page AS Cattani L Pacquée S Claerhout F Callewaert G Housmans S. Long-term data on graft-related complications after sacrocolpopexy with lightweight compared with heavier-weight mesh. Obstet Gynecol. (2023) 141(1):189–198. 10.1097/AOG.0000000000005021 36701619

[16] Pycek M Zarzecka JM Majkusiak W Barcz EM Zwierzchowska A. Current approach to the use of transvaginal mesh systems in pelvic organ prolapse. Ginekol Pol. (2025) 96(5):414–419. 10.5603/gpl.101432 40145699

[17] FDA News Release. FDA takes action to protect women’s health, orders manufacturers of surgical mesh intended for transvaginal repair of pelvic organ prolapse to stop selling all devices. U.S. Food and Drug Administration. (2019).

[18] Sun MJ Chuang YL Lau HH Lo TS Su TH. The efficacy and complications of using transvaginal mesh to treat pelvic organ prolapse in Taiwan: a 10-year review. Taiwan J Obstet Gynecol. (2021) 60(2):187–192. 10.1016/j.tjog.2021.01.031 33678316

[19] Liang S Guo J Zhu L. 11601 Complications and efficacy after transvaginal mesh repair for Chinese women in the primes registry. J Minim Invasive Gynecol. (2024) 31(11):S94–5. 10.1016/j.jmig.2024.09.788

[20] Maher C Feiner B Baessler K Christmann-Schmid C Haya N Marjoribanks J. Transvaginal mesh or grafts compared with native tissue repair for vaginal prolapse. Cochrane Database Syst Rev. (2016) 2(2):CD012079. 10.1002/14651858.CD012079 26858090 PMC6489145

[21] Deffieux X Perrouin-Verbe MA Campagne-Loiseau S Donon L Levesque A Rigaud J. Diagnosis and management of complications following pelvic organ prolapse surgery using a synthetic mesh: french national guidelines for clinical practice. Eur J Obstet Gynecol Reprod Biol. (2024) 294:170–179. 10.1016/j.ejogrb.2024.01.015 38280271

[22] Gold RS Neuman J Neuman M Groutz A. Size does matter: long-term efficacy and safety of minimesh in transvaginal pelvic organ prolapse repair. Gynecol Minim Invasive Ther. (2026) 15(1):30–35. 10.4103/gmit.GMIT-D-24-00016 41797946 PMC12965510

[23] Barski D Arndt C Gerullis H Yang J Boros M Otto T. Transvaginal PVDF-mesh for cystocele repair: a cohort study. Int J Surg. (2017) 39:249–254. 10.1016/j.ijsu.2017.02.006 28192248

[24] Shin EJ. Surgical treatment of rectal prolapse. J Korean Soc Coloproctol. (2011) 27(1):5–12. 10.3393/jksc.2011.27.1.5 21431090 PMC3053504

[25] O'Connor A Byrne CM Heywood N Davenport M Klarskov N Sharma A. Anal sphincter function in rectal intussusception and high and low “take-off” external rectal prolapse-A prospective observational study. Colorectal Dis. (2024) 26(12):2069–2079. 10.1111/codi.17191 39370561 PMC11649865

[26] Wells CA. A new operation for rectal prolapse. Proc R Soc Med. (1959) 52(8):602–603. 10.1177/003591575905200805 13843894 PMC1870053

[27] Frykman HM Goldberg SM. The surgical treatment of rectal procidentia. Surg Gynecol Obstet. (1969) 129(6):1225–1230. 5353418

[28] Li Z Xu T Li Z Gong J Liu Q Zhu L. An epidemiologic study of pelvic organ prolapsein rural Chinese women: a population-based sample in China. IntUrogynecol J. (2019) 30(11):1925–1932. 10.1007/s00192-018-03859-9 30685785

[29] Umeh UA Ugwu EO Obi SN Nnagbo JE. Concurrent occurrence of uterovaginal and RP: an uncommon presentation. Ann Med Health Sci Res.2015:5(5):365–367. 10.4103/2141-9248.165256 PMC4594351 26500795

[30] Karatake A Batu P Asoglu MR Selcuk S Cam C. Approach to concomitant rectal and uterine prolapse: case report. J Turk Ger Gynecol Assoc. (2012) 13(1):70–73. 10.5152/jtgga.2011.67 24627680 PMC3940228

[31] Zhang XL Lu YX Shen WJ Zhao Y Niu K Wang WY. Safety and long-term efficacy of transvaginal reconstructive pelvic surgery for severe pelvic organ prolapse in elderly women aged 70 years and over. Zhonghua Fu Chan Ke Za Zhi. (2025) 60(8):627–636. 10.3760/cma.j.cn112141-20250220-00058 40854748

[32] Chen J Yu J Morse A Tao G Gong J Wang B. Effectiveness of self-cut vs mesh-kit Titanium-coated polypropylene mesh for transvaginal treatment of severe pelvic organ prolapse: a multicenter randomized noninferiority clinical trial. JAMA Netw Open. (2022) 5(9):e2231869. 10.1001/jamanetworkopen.2022.31869 36112377 PMC9482053

